# Community mobilisation and health management committee strengthening to increase birth attendance by trained health workers in rural Makwanpur, Nepal: study protocol for a cluster randomised controlled trial

**DOI:** 10.1186/1745-6215-12-128

**Published:** 2011-05-19

**Authors:** Joanna Morrison, Kirti Man Tumbahangphe, Bharat Budhathoki, Rishi Neupane, Aman Sen, Kunta Dahal, Rita Thapa, Reema Manandhar, Dharma Manandhar, Anthony Costello, David Osrin

**Affiliations:** 1 Centre for International Health and Development, Institute of Child Health, University College London, 30 Guilford Street, London, WC1N 1EH, UK; 2 Mother and Infant Research Activities, PO Box 921, Thapathali, Kathmandu, Nepal

## Abstract

**Background:**

Birth attendance by trained health workers is low in rural Nepal. Local participation in improving health services and increased interaction between health systems and communities may stimulate demand for health services. Significant increases in birth attendance by trained health workers may be affected through community mobilisation by local women's groups and health management committee strengthening. We will test the effect of community mobilisation through women's groups, and health management committee strengthening, on institutional deliveries and home deliveries attended by trained health workers in Makwanpur District.

**Design:**

Cluster randomised controlled trial involving 43 village development committee clusters. 21 clusters will receive the intervention and 22 clusters will serve as control areas. In intervention areas, Female Community Health Volunteers are supported in convening monthly women's groups. The groups work through an action research cycle in which they consider barriers to institutional delivery, plan and implement strategies to address these barriers with their communities, and evaluate their progress. Health management committees participate in three-day workshops that use appreciative inquiry methods to explore and plan ways to improve maternal and newborn health services. Follow-up meetings are conducted every three months to review progress. Primary outcomes are institutional deliveries and home deliveries conducted by trained health workers. Secondary outcome measures include uptake of antenatal and postnatal care, neonatal mortality and stillbirth rates, and maternal morbidity.

**Trial registration number:**

ISRCTN99834806

## Background

### Maternal and child mortality

The 4^th ^and 5^th ^Millennium Development Goals (MDGs) aim for reductions in child mortality and maternal mortality. Recent data suggest that only 23 out of 81 countries are on track to reduce Maternal Mortality Ratios (MMRs: maternal deaths per 100 000 live births) by 75% [1]. Progress towards MDG 4 has also been suboptimal, with only 16 of 68 target countries on track to meet under-five mortality reduction targets[2]. Ensuring skilled attendance in a safe delivery place is an important strategy to increase both newborn and maternal survival[3]. In the poorest countries, most deaths of mothers and newborns occur in the home, many women deliver without trained health workers and access to quality care is limited. Research is necessary to enable evidence-based decision-making about how to address barriers to accessing care, and improvement of health services through a cost effective, sustainable and scaleable approach.

### Community mobilisation for health

Community mobilisation strategies to improve health outcomes became popular through the primary health care movement[4]. Renewed interest in this approach has led researchers and policy makers to seek impact evaluations in order to make evidence-based decisions[5]. There are different models of community mobilisation. Some engage community members as passive recipients of a programme, similar to the expanded programme on immunisation, and others seek active participation of target populations[6]. Participatory action cycle approaches are often used in community mobilisation. The approach is problem focussed, and the process begins by individuals coming together to develop an understanding of the problem and its causes. This group continues to work together with other stakeholders to address the problem and initiate social change. The actions taken are then critically examined and the cycle continues through iteration [7,8]. A study in Bolivia, the Warmi project, provided some evidence that increasing participation of communities in a maternal and newborn health project could improve health outcomes[9,10]. Our research collaboration between Nepal's Mother and Infant Research Activities (MIRA) and University College London (UCL) adapted this intervention and conducted a cluster randomised controlled trial to evaluate its impact on newborn mortality[11,12]. We reported a 30% reduction in newborn mortality in intervention areas when compared with control clusters[13]. We also found increased cleanliness at birth, care-seeking during illness, and significant positive differences in institutional delivery and trained birth attendance. A similar community-based cluster randomised controlled trial with research partners in rural India also found significant decreases in newborn mortality. The impact of the intervention increased over time, and it had positive effects on postnatal depression and women's agency[14]. Process evaluations suggest that the participatory action oriented approach was key to developing the capacity of communities to address problems, and enabling community empowerment[15,16]. Other cluster randomised controlled trials of community mobilisation interventions have not shown reductions in mortality, but have shown other benefits to communities[17,18].

### Recent developments in Nepal's health sector

Maternal and newborn health are improving in Nepal, demonstrated by a drop in maternal mortality ratio from 539 per 100 000 live births in 2001 to 281 in 2006. Despite these improvements, most women still give birth at home without a trained health worker. In response, the Government of Nepal (GoN) has developed a national policy on skilled birth attendance (SBA) and a national in-service training strategy. The GoN has set high targets of 60% of deliveries with a skilled attendant by 2012 and recruitment and training of over 4000 health personnel. In order to encourage families to access safe delivery services, the government introduced a maternity incentive scheme in 2005, providing cash in hand to cover the travel costs of delivery in a health institution [19]. The incentive varies from NRs 500 (US$7) in the plains to NRs 1500 (US$21) in the mountains. Free delivery care was introduced in districts with a low human development index [20], and was rolled out nationally at the beginning of 2009 [21]. Our estimate of institutional deliveries in the rural population of Makwanpur District where our trial is being conducted is 17%.

### Female Community Health Volunteers

Complementary strategies for newborn and child health are the national community based Integrated Management of Childhood Illness strategy (IMCI) [22], and the implementation of a community-based newborn care package in 10 districts [23]. An important cadre in both of these is the Female Community Health Volunteer (FCHV). Around nine FCHVs per village development committee unit (VDC) are responsible for convening women's groups, conducting community level health education and providing services such as immunisation for tetanus and polio, distribution of iron and folic acid tablets and Vitamin A, oral rehydration and family planning. They are nominated by their local women's group and receive 18 days initial training on primary health care, and five days of refresher training per year. FCHVs are not salaried, but are given access to micro-credit funds and allowances for attending training. The GoN has issued guidelines for a daily allowance of NRs 200 (US$3) for them to attend meetings or training with non-governmental organisations. VDCs may also provide a financial or non-financial incentive at their discretion. These may include the provision of a uniform, umbrella or bicycle [24]. The success of national primary health care programmes has been credited to the key role of the FCHV [25], and she may be an important link between communities and health services. She is often the first point of referral for mothers and children.

Operational research suggests that FCHVs may be effective in treating and referring newborn infections if they are supported by an organisation or a well-functioning health system [26-28]. We are conducting a cluster randomised controlled trial to test the effectiveness of FCHVs in the treatment of newborn sepsis, and community mobilisation through women's groups on neonatal mortality, in sub-optimal health system conditions [29]. Formative research in Makwanpur district found that FCHVs lacked skills in participatory discussion, and it was difficult for them to sustain regular women's group meetings without regular training, supervision, an agenda and tools to facilitate discussions [30].

### Health management committees

**Figure 1:**
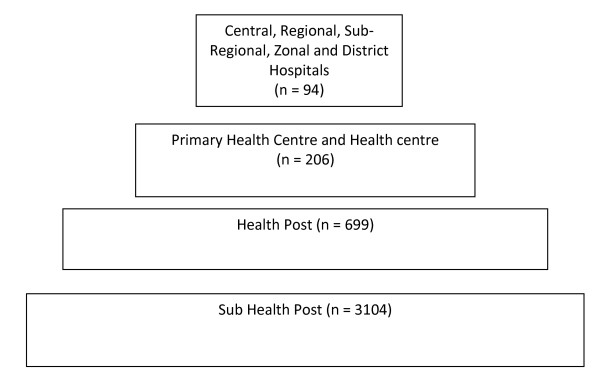
**Government of Nepal Health Care Facilities**.

The sub-health post (SHP) is the most basic level of institution in Nepal's health system pyramid, followed by the health post (HP), primary health care centre (PHC), and Hospital (Figure 1) [31]. SHPs and HPs are expected to provide first level obstetric care. They should stabilise maternal problems with obstetric first aid, make appropriate referral and arrange transport. PHCs should provide basic emergency obstetric care (BEOC), and comprehensive essential obstetric care (CEOC) should be provided at district, regional and zonal hospitals. Each VDC has at least one health institution, and each health institution has a management committee (HMC). HMCs should meet at least once a month, and meet with the hospital quality of care committees three times a year to develop needs-based plans, audit the quality of care and take steps to improve it [32]. HMCs are usually chaired by the VDC political representative and include the health facility in-charge and community representatives. It is recommended that one of these representatives be from a minority (Janajhati), ethnic group and another from a lower caste (Dalit) group. The involvement of social, or community workers (such as school teachers, or community members involved in development activities) are also recommended, and a Female Community Health Volunteer is usually a member of the HMC. The activity of HMCs varies [33], and has been hindered in recent years by the dissolution of local political structures. The present GoN policy to strengthen local governance and increase decentralisation of decision-making to VDCs has led to increased opportunities for health service improvements [34].

Substantial improvements in maternal and newborn health are not possible in the context of weak health systems [3,35]. A synergistic approach is necessary to address issues of demand and supply [36]. Involving communities in planning and implementing health services while strengthening health systems can increase accountability, enable the development of responsive, appropriate services and increase uptake and awareness of the need for care. Health systems improvement strategies have been influenced by organisational management theory and practice. In particular, Appreciative Inquiry approaches have been utilised to stimulate organisational change in the health context [37-39]. Appreciative inquiry challenges the problem-solving approach, suggesting that highlighting the weaknesses of an organisation may lead to a 'degenerative spiral' [40]. Instead of focusing on the problems, organisations are invited to identify and become conscious of the 'good', enabling them to envision what is possible, instead of what is wrong [41]. This approach enables participants to reflect on existing strengths, discover what is important and build a collective vision of a shared future [38]. Although Appreciative Inquiry has become popular, published outcome evaluations are scarce. A review of the literature in 2004 [42] identified only three empirical studies[43-45].

The Nepal Safer Motherhood Project used an Appreciative Inquiry approach to assist staff to "create new possibilities, new visions, and to take responsibility for creating and initiating changes" [46]. This strategy was also utilised with health management committees as part of the later Support for Safe Motherhood Programme. A process evaluation of 12 facilities at an early stage of the intervention found some positive changes in the availability of female trained health workers, some infrastructure improvements, and the availability of 24-hour delivery care. The report cited a need for monitoring, technical support, and follow-up as critical to sustaining motivation and momentum for change [47]. Another recent descriptive study also identifies a need to maintain inputs to sustain and develop institutional change in HMCs [48].

### Trained birth attendants

In the 1990s Nepal invested in two cadres of health workers to provide maternal and child health services and obstetric first aid at village level: Maternal and Child Health Workers (MCHWs) and Auxiliary Nurse Midwives (ANMs). Unfortunately, neither cadre functioned successfully as trained birth attendants. The reasons for this included limited duration of training, lack of competency-based training, lack of clinical experience, professional and social isolation in post, and lack of support from the health system to enable them to provide quality emergency obstetric and neonatal care [49]. Both internationally and nationally, there is some debate about the nature of SBA [50,51]. In order to claim the requisite 'skills', a health care provider should have been trained specifically in BEOC and should also have had sufficient clinical exposure to be able to competently put her training in practice. The GoN defines a skilled birth attendant as "an accredited health professional - such as a midwife, doctor or nurse - who has been educated and trained to proficiency in the skills needed to manage normal (uncomplicated) pregnancies, childbirth and the postnatal period and in the identification, management and referral of complications in women and newborns" [49,50]. In 2004, it was agreed that, in the context of Nepal, doctors, staff nurses, midwives and ANMs would be considered skilled birth attendants, provided that they possess competencies in specified core skills [50].

Most deliveries occur without a skilled attendant, and although there is political will to increase skilled attendance at delivery, it will take time to recruit and train an adequate workforce. The recent maternal mortality and morbidity study found that implementation of quality care was dependent on the availability of trained staff, as opposed to geographical region or district. There are eight current strategies for increasing SBA in Nepal: (1) human resource development, (2) strengthening SBA training sites, (3) deployment and retention of skilled attendants, (4) service provision, (5) an enabling environment, (6) support from professional organizations, (7) support from the non-government and private sectors, particularly through establishing maternity hospitals and community birthing centres, and (8) institutional arrangements within the Ministry of Health and Population[52]. A national in-service training strategy for SBA has also been drafted[53]. Medical officers, staff nurses, and ANMs working at PHCs and health posts have been prioritised to receive SBA training [54].

### Study objectives

#### Purpose

We aim to test the effect on institutional deliveries and home deliveries by trained health workers of a combination of community mobilisation through women's groups and strengthening of health management committees.

#### Primary research question

Will a combination of community mobilisation through women's groups and strengthening of health management committees lead to increased birth attendance by trained health workers, both at home and in health institutions?

#### Secondary research questions

Will the complex intervention increase uptake of antenatal and postnatal care?

Will the intervention increase access to national programmes of free delivery care and government incentives?

Through a plausibility analysis, we will also examine the effects of the intervention on maternal morbidity and mortality and newborn mortality.

### Design

**Figure 2:**
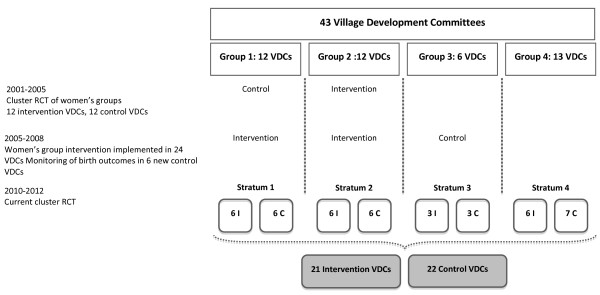
**Trial cluster allocation process based on previous project activities**.

The effectiveness of the intervention is being tested through a cluster randomised controlled trial. Because the intervention is targeted at communities, the unit of allocation is the VDC cluster. We randomly allocated the 43 VDCs in the district to receive the intervention (n = 21) or serve as control areas (n = 22). We have already worked in 30 VDCs in Makwanpur district to evaluate the effectiveness of women's group interventions, and we felt it was important to stratify sampling on the basis of previous exposure of clusters to programme activities. The success of these activities had led to measurable differences between VDCs. We stratified clusters into four groups. Group 1 included control clusters from 2002-2005, and intervention clusters from 2005 to 2008. Group 2 included intervention clusters from 2001-2008. In group 3 we monitored birth outcomes from 2005-2008. In group 4, we had not previously conducted any intervention or monitoring activities. We randomly allocated equal numbers of clusters from each group to intervention and control clusters using the methods detailed below. Although the cluster number is large, and it is possible that unstratified allocation would lead to a balanced design, we preferred a stratified design because of its intelligibility to residents of the district (see Figure 2).

We considered the issue of contamination. Within each rural VDC, houses tend to be clustered in scattered settlements. Each VDC has one health institution: a health post or primary health centre. Not all of these offer institutional delivery. It is therefore possible that a woman might cross into another VDC for her delivery (historically, since institutional delivery was rare, the crossover tended to be either to the district hospital in the municipality of Hetauda or to a large hospital in another district entirely). The intervention was not designed to increase trained birth attendance through institutional delivery at a specific health facility. We seek only to increase such deliveries at any appropriate institution. One means to do this will be the activities of women's groups, which are specific to individually allocated VDCs. Health Management Committees will operate around the institutions specific to their VDCs, but may not in all cases aim to increase deliveries at their own institutions. Where institutional delivery is not possible, the blanket aim is to put processes in place to ensure that local residents make the right choice of site so that they have a safe delivery. For contamination to occur, either the influence of women's group activities must disseminate beyond the VDC (push factor), or the influence of HMC activities must encourage attendance at their institutions (pull factor). On the basis of our experience during the previous trial of women's groups, we do not think that the former is likely to be a strong effect. The latter is a possibility, which would tend to reduce the difference between intervention and control arms of the trial.

#### Allocation

Allocation was conducted at a meeting in Hetauda, the district centre, chaired by a member of the MIRA executive committee. We invited the Local Development Officer (as chief guest), the District Health Officer, the District Public Health Officer, the chair of the district Non Governmental Organisation (NGO) federation, the Women's Development Officer, representatives of several political parties, and heads of health sector NGOs. A presentation was made on the history of the programme and the design of the new trial. Previous experience suggested that local stakeholders found it easy to understand randomised controlled trials through an analogy with a lottery. A lottery apparatus was used, with the pre-coded number of each village development committee written onto an individual ball. At this stage, participants did not know the number of each area. Balls were loaded into the lottery apparatus according to strata and selected by the chief guest. The first six balls selected in the first stratum were allocated to the intervention, and so on across the four strata. When all 21 balls representing allocation to the intervention had been selected, the names and allocation of the village development committees were read out. We invited major stakeholders to give short speeches about their perceptions of the process and the chief guest closed the meeting. The event was documented with video and still photographs, available on request.

#### Primary outcome measures

1. Deliveries conducted by trained health workers.

2. Institutional deliveries.

#### Secondary outcome measures

1. Antenatal care uptake.

2. Postnatal care uptake.

3. Neonatal deaths per 1000 live births.

4. Stillbirths per 1000 births.

5. Maternal morbidities.

Skilled birth attendance is an internationally accepted indicator of the success of interventions to improve maternal health [3,55]. This is mainly because it is thought to be on the causal pathway, and because maternal mortality is a sufficiently rare outcome to prevent its use in trials of this nature. Institutional deliveries are a subset of deliveries conducted by a skilled attendant. Our intervention targets health systems and communities. The causal pathway to improved outcomes includes uptake of antenatal and postnatal care services and improved management of maternal morbidities. Previously, our intervention sought to improve neonatal survival, and it will be interesting to examine if further neonatal gains can be made with increased utilization of services.

## Methods

### Setting and formative work

The trial is being conducted in rural Makwanpur District, south of Kathmandu. Makwanpur is topographically diverse with plains and hill areas, covering 2500 km^2^. It has a population of around 400 000 and a Human Development Index of 0.48. 36% of the population fall below the poverty line of $1 a day per household[56]. The female literacy rate is 54% and the male rate 73%. The district centre of Hetauda has a hospital in which there are around 117 deliveries per month, and one private hospital that offers delivery services. There are ten HPs in the district, of which seven are birthing centres, and 30 SHPs, of which eight are birthing centres. There are four PHCs with seven doctors in the district, ten staff nurses (four in PHCs), and 55 ANMs. Health Management Committees recruited 21 of these ANMs. Two staff nurses and nine ANMs in the district have received SBA training. Our research suggests a current trained birth attendance level of 17% in rural VDCs, up from about 5% in 2000. There are plans to provide CEOC services at Hetauda hospital, but this was not the case at the time of writing. Our health facility audit found that the supply of essential drugs for maternal and newborn care was adequate, although drugs to manage pre-eclampsia and eclampsia were often unavailable in birthing centres. Delivery sets, newborn resuscitation and thermal care facilities were not routinely available, despite the fact that 19 facilities are designated birthing centres. In addition, many SHPs did not have a toilet or running water, and a constant supply of light was not available in 50% of health institutions. GoN Family Health Division Support to the Safe Motherhood Programme conducted a district level workshop about the roles and responsibilities of the HMC, in particular their role in ensuring the provision of safe motherhood services. This was conducted in the district centre of Hetauda in January 2010, and political representatives, health facility representatives, and some HMC members participated. A two-day follow-up programme was also organised. Our formative research found that only 15 out of 43 HMCs had participated in an orientation programme. HMC meetings were not held on a regular basis and were often postponed because of an absence of committee members, particularly political party representatives. 17 HMCs had initiated staff recruitment within the year preceding our formative study, and 12 had taken steps to fill vacant posts. To get a feel for the functionality of HMCs, we categorised a well-functioning HMC as one that had reported initiating four or more activities in the year preceding the study, and found that 18 out of 43 HMCs met our criteria. We found that 90% of HMC funding comes from the VDC and some HMCs are taking advantage of opportunities to allocate resources to local priority areas.

### Target Population

Women of reproductive age, family members, health service cadres, health management committee members, and communities. We estimate conservatively that there will be over 4000 pregnancies per year in the study area.

### Sample size

The study will include all pregnancies in all 43 VDCS. We estimate that each VDC will yield about 100 births per year and that the proportion of birth attendance by trained health workers is currently 20%. We estimated sample size using the equations of Hayes and Bennett [57], assuming two treatment groups and unmatched clusters of approximately equal size. We set a value of *k *- the between-cluster coefficient of variation - equal in intervention and control groups, and added 2 to the estimated cluster number to account for loss of degrees of freedom as a result of stratification. All estimates are based on a two-tailed 5% significance level. The value of *k *is likely to be 0.3 on the basis of estimates of neonatal mortality rate from the Makwanpur study. However, we have estimated sample size with a range of *k *from 0.25 to 0.35.

For two years of intervention (200 births per cluster), if *k *= 0.35, 42 clusters would detect an increase in trained attendance from 20% to 30% at 90% power, and from 20% to 28% at 80% power. If *k *= 0.30, 42 clusters would detect an increase in trained attendance from 20% to 28% at 90% power, and from 20% to 27% at 80% power. If *k *= 0.25, 42 clusters would detect an increase in trained attendance from 20% to 27% at 90% power, and from 20% to 26% at 80% power. Overall, a conservative level of powers, births and values of *k *suggests that the sample size will be able to detect an increase in birth attendance by trained health workers of around 10% over two years.

### The intervention

The intervention being implemented in 21 clusters has two components: community mobilisation through women's groups, and strengthening of health facility management committees. Previously, our intervention employed a cadre of local female facilitators to lead groups through a participatory action cycle about maternal and newborn health [8,11]. Although it was successful in reducing newborn mortality, most women in the study gave birth at home without a trained health worker in attendance [13]. Qualitative research found that there was often lack of trust in local health facilities and contact between women's groups and health institutions was limited [15]. In dialogue with the GoN Family Health Division, we sought to develop a model of community mobilisation that was scaleable within existing government health systems, was more focussed on enabling trained health worker attendance at deliveries, and could enable links to be developed between communities and health facilities. We have developed an intervention that fits national policy by engaging FCHVs to facilitate discussions on maternal and newborn health in their regular monthly women's group meetings. Our model of support and supervision is less intensive than in previous studies, with a lower ratio of FCHVs to supervisors, increasing the potential for scale-up within existing systems. We are also conducting HMC strengthening through an Appreciate Inquiry approach endorsed by the Family Health Division [40,58]. We aim to increase links between communities and health institutions and improve access to quality maternal and newborn health services.

#### Women's groups

195 FCHVs received four days training to orientate them about our previous research in Makwanpur, familiarise them with the participatory action oriented approach of the intervention and develop their facilitation and reporting skills. They received refresher training on their roles and responsibilities and were introduced to the manual they will use to help guide discussions. Formative data show that 50% of FCHVs in the intervention area had difficulty in reading and writing. The manual contains a simple discussion guide for each meeting, stating its objectives and encouraging the use of tools or games. FCHVs are supervised by seven MIRA field supervisors who co-ordinate monthly orientation meetings in each VDC to discuss progress, problems, and the next women's group meeting agenda. Supervisors also support FCHVs while they are conducting meetings, observing an average of four meetings per month. Supervisors meet with a Facilitation Manager once a month to report on progress and discuss issues from the field. 203 women's groups are led through a participatory action cycle. In the first seven meetings groups are encouraged to interact with their community in identifying barriers to trained health worker attendance at deliveries, and then to develop ways of addressing them. Groups then have a community meeting where they present and discuss the barriers and corresponding strategies to deal with problems, seeking support and commitment from community members. After the community meeting, groups develop plans and lead the implementation of strategies to address problems. This phase takes place without a discussion manual and has no set duration. After around four months, groups will be encouraged to evaluate the strategies they have implemented with the aid of a manual, and plan the next steps. We will complete two cycles of problem identification, planning, implementation and evaluation within the study period.

#### Health Management Committee strengthening

We developed the intervention after discussions with the Support for Safe Motherhood Programme, United Mission to Nepal, and Appreciative Inquiry practitioners in India and Nepal. The intervention uses the principles of the 'four D' cycle of discovery, dream, design and destiny [40]. We also visited HMCs that had engaged in appreciative planning and action in order to learn from their experience. We employed a consultant to conduct a training of trainers on the Appreciative Planning and Action process with MIRA researchers, representatives from the District Public Health Office, District Development Committee, and Family Planning Association of Nepal. Thereafter, we conducted four-day workshops in each of the 21 intervention VDCs over four months. Workshops were held in local health facilities and attended by a district level representative who had attended the training of trainers. A senior MIRA researcher facilitated each workshop. HMC members, community representatives, and health facility employees participated. Our formative research found 18 out of 43 HMCs scored well on indicators of resource mobilisation, staff management, service monitoring, management of health volunteers, and regularity of meetings. 10 were in control areas, and eight in intervention areas. We also found that many HMCs had not received an orientation about their roles and responsibilities, and we discussed this on the first day of the workshop. We familiarised participants with the maternal and newborn health situation in Nepal, government strategies and priorities, and our research. After briefing participants about the Appreciative Inquiry methodology and philosophy using stories, reports and proverbs, participants were invited to 'discover' the successes of their health institution and remember who provided support or resources to facilitate this success. Participants then 'dreamt' of how they would like their health institution to be and the quality of services they would like to provide for maternal and newborn health. In the next phase they 'designed' a strategy to achieve their vision, using resources and opportunities discussed in the discovery phase, detailing goals and strategies to reach them, and allocating responsibilities for activities. We will support and monitor the progress of the HMCs through follow-up meetings every two to three months. These discussions will encourage HMCs to track their progress on strategy implementation and add or change plans as necessary. The last phase of the appreciative planning and action approach, 'destiny', will be completed after HMCs have implemented their plans, and participants will present their accomplishments and what they have learned.

### Data collection

Data on the primary outcomes will be collected through a surveillance system for all births in the 43 VDCs. The system is similar to those implemented in other community based trials[14,17,59-61]. We incentivise nominated local women to identify births, newborn deaths and deaths of women between the ages of 12 and 49 years. They communicate vital event information at monthly meetings to a cadre of interviewers who verify the events and visit families to administer a questionnaire. Questionnaires to measure our primary and secondary outcomes are based on previous tools. We piloted 100 questionnaires and adapted the tool based on discussions with our experienced team of interviewers. Standard WHO verbal autopsy tools were translated from English to Nepali, back translated and field tested, and are used to ascertain cause of death [62].

### Project Timeline

Prospective baseline enrolment of births: 19^th ^November 2009 - 30^th ^September 2010.

Enrolment of births in the trial: 1^st ^October 2010 - 30^th ^September 2012.

Women's group meetings: March 2010 - 30^th ^September 2012.

Health management committee strengthening workshops: June 2010 - 30^th ^September 2012.

Health management committee strengthening follow-up: December 2010 - September 2012.

Intervention duration: 1^st ^October 2010 - 30^th ^September 2012.

### Data management

Questionnaire responses will be checked in the clusters by field coordinators, and re-checked by a data auditor before data entry in the district office in Hetauda. At least 10% of interviews will be observed. Information will be entered in a relational database management system (RDBMS) in Microsoft SQL Server 2007, through an interface programmed in Visual Basic for Applications. The system will incorporate validation constraints. Hard copies of records will be stored in a filing system in a lockable room. Electronic output will be anonymised.

We are also conducting a concurrent process evaluation to describe the context of the intervention, how it is being implemented and who is participating, and collecting information to develop hypotheses about how the intervention may be effective. The integration of process evaluations with randomised controlled trials enables the external validity of the trial findings to be assessed, [63] and may help in successful replication [64]. Process data also help understand and explain trial results [65]. Qualitative and quantitative data are being collected, entered and analysed by our process evaluation team who have received training and support in qualitative and quantitative techniques.

#### Interim analysis

We will convene a Data Monitoring Committee in 2012 after 18 months of project activities. The board will include national and international expertise in the conduct of randomised controlled trials and will review data and procedures according to the DAMOCLES guidelines [66]. The committee will be decide whether the study protocol has been followed adequately, whether enrolment has been adequate, and whether randomization has resulted in comparable groups so that, in the absence of interventions, it would be reasonable to expect similar health outcomes over time. It will review interim data for completeness, quality and adherence to ethical requirements, and will make recommendations on the continuation of the trial or its modification.

#### Analysis strategies

Data will be checked for consistency and completeness in both the RDBMS and after export to Stata. An analysis plan will be developed in accordance with the CONSORT reporting guidelines [67]. We will present a trial profile and a comparison of key variables between intervention and control arms at baseline. Details of the number of clusters and individuals included in the analysis will be provided, and the analysis will be intention-to-treat. We will compare the primary outcome in intervention and control arms, using data for two years of intervention. We will conduct the analysis at the level of the individual, and the procedure of choice will be random effects logistic regression models grouped on cluster. If quadrature checks suggest that this is inappropriate, we will use generalised estimating equations. Analyses will be adjusted for socioeconomic status and religion. The findings will be augmented by ancillary analyses, which may include dose-response, interrupted time series and process evaluations.

### Ethical issues

The study was approved by the Nepal Health Research Council on 12^th ^February 2009, and the University College London Ethics Committee on 13^th ^September 2010.

Before allocation, we conducted community meetings in each VDC cluster, usually in the health facility building, school or community hall. We invited the chairperson of the HMC, representatives of local and international non-governmental organisations, community based organisations, teachers, FCHVs, political leaders, health personnel, political representatives, and social leaders. We aimed to have at least 20 people present at each community meeting and between 17 and 49 people attended. We presented our previous research and current research plan, our objectives and the utility of the research using graphs and pictures for ease of understanding. There followed an open discussion, question and answer session, and written consent to conduct the study was taken at the cluster allocation meeting. In addition, interviewers approach respondents in their homes, provide information and then take verbal consent before conducting interviews. After trying to obtain written consent with some respondents who were literate, we decided that, for consistency, it would be better to take informed verbal consent in this largely illiterate population. Participation in the HMC or women's group intervention is voluntary and based on personal interest. Participants are free to opt in or out as they wish.

#### Benefits to control areas

During previous research in the district, we have given Essential Newborn Care training to all health personnel and FCHVs, and have provided basic equipment for newborn care in health facilities. In the current trial, we will train all health workers in the district in Obstetric First Aid and re-supply essential newborn care equipment. This ensures a minimum level of care in the study area.

#### Sustainability and scalability

We are working with the government cadre of the FCHV to implement the women's group intervention, which increases the sustainability of our approach. We found that 77% (n = 183) of groups mobilised in previous interventions were continuing to meet 12 months after MIRA stopped supporting them. Half of these groups were being facilitated by the FCHV and a group member ran the other half. 60% of groups were confident that they would continue meeting for the next two years[68]. This suggests community ownership and integration, and increased potential for the intervention and its effects to endure beyond the trial.

We have trained district level health personnel in an Appreciative Planning and Action approach and they have facilitated sessions at every workshop with HMCs. Political representatives, health personnel, and community members participated in HMC strengthening workshops, with district level employees, creating linkages and strengthening communication between local and district level personnel. Through strengthening existing local structures of FCHV-led women's groups and HMCs, we seek to enable sustained linkages between health facilities and communities. We hope to enable communities to maintain improved services by building local capacity and commitment to change.

### Public engagement

We will promote public engagement in our research locally, nationally and internationally.

Locally, we have been engaged in dialogue with community representatives, political bodies, community-based organisations (CBOs) and NGOs since cluster allocation. Intervention areas will have formal community meetings between women's groups and community members after seven months and at least once more during the intervention period. MIRA employees will be present at these meetings. After completion of the study, we will disseminate the results formally at meetings for all VDCs of Makwanpur district. We will invite VDC representatives, health management committee members, health post in-charges, teachers, user group members, NGOs, CBOs, local intellectuals, political party representatives, cooperative members, VDC secretaries, club members and local residents. After presenting the findings, the forum will be open for discussion and comment and feedback will be taken. Likewise, we will disseminate the findings at district level. The District Health Officer, District Public Health Officer, Local Development Officer, Community Development Officer, Regional Administrator, Women's Development Officer, Municipality Chief and representatives of International NGOs and NGOs working in the women's and children's sectors will be invited to the meeting. We will engage in open discussion at these meetings.

Nationally, we consulted the GoN Family Health Division prior to seeking ethical approval for the study, and continue to discuss the study with them. We have learned from GoN experience with health management committee strengthening, and have shared experience and tools to implement emergency obstetric first aid training in Makwanpur. We hope that by actively engaging the GoN in our research they will use our findings to inform the development of policy for maternal and newborn health. We will disseminate our findings to policy makers, programme managers and representatives of international organizations working in Nepal. We will also present our research at national conferences of professional bodies, for example the Perinatal Society of Nepal (PESON), Nepal Society of Obstetricians and Gynaecologists (NESOG), Nepal Pediatric Society (NEPAS) and the Nepal Medical Association (NMA), and at Tribhuvan University. We will publish relevant aspects of the study in national medical journals associated with these professional bodies. We will also summarise research findings on the MIRA website.

Internationally, we will publish articles in peer-reviewed journals, which have a history of influencing health policy. Through connections facilitated by a Wellcome Trust Strategic Partnership, we will disseminate the findings and recommendations to public health academics, policymakers, bilaterals and international NGOs. Our Strategic Partnership will build a communications strategy and seek resources to disseminate and engage the public in our research findings. We will engage stakeholders through policy briefs, conferences, workshops and websites.

## Abbreviations

ANM: Auxiliary Nurse Midwife; BEOC: Basic Emergency Obstetric Care; CBO: Community Based Organisation; CEOC: Comprehensive Essential Obstetric Care; CONSORT: Consolidated Standards of Reporting Trials; DAMOCLES: Data Monitoring Committees: Lessons, Ethics, Statistics; FCHV: Female Community Health Volunteer; GoN: Government of Nepal; HMC: Health Management Committee; HP: Health Post; IMCI: Integrated Management of Childhood Illness; MCHW: Maternal and Child Health Worker; MDG: Millennium Development Goals; MIRA: Mother Infant Research Activities; MMR: Maternal Mortality Ratio, maternal deaths per 100 000 live births; NEPAS: Nepal Paediatric Society; NESOG: Nepal Society of Obstetricians and Gynaecologists; NGO: Non Governmental Organisation; NMA: Nepal Medical Association; NRs: Nepali Rupees; PESON: Perinatal Society of Nepal; PHC: Primary Health Care Centre; RDBMS: Relational Database Management System; SBA: Skilled Birth Attendant; SHP: Sub Health Post; UCL: University College London; VDC: Village Development Committee; WHO: World Health Organisation.

## Competing interests

The authors declare that they have no competing interests.

## Authors' contributions

JM and DO wrote the first draft of the paper, contributed to the study design and analysis plan, and are technical advisors to the study. All authors reviewed the manuscript and commented on drafts of the paper. AC and DM are principal investigators. AS, BB, KD, KT, RN, RM and RT, manage and implement the trial.
